# The truth lies somewhere in the middle: the cells responsible for liver tissue maintenance finally identified

**DOI:** 10.1186/s13619-021-00090-8

**Published:** 2021-08-04

**Authors:** Tohru Itoh

**Affiliations:** Laboratory of Stem Cell Therapy, Institute for Quantitative Biosciences (IQB), The University of Tokyo, 1-1-1-Yayoi, Bunkyo-ku, Tokyo, 113-0032 Japan

## Background

**Fig. 1:**
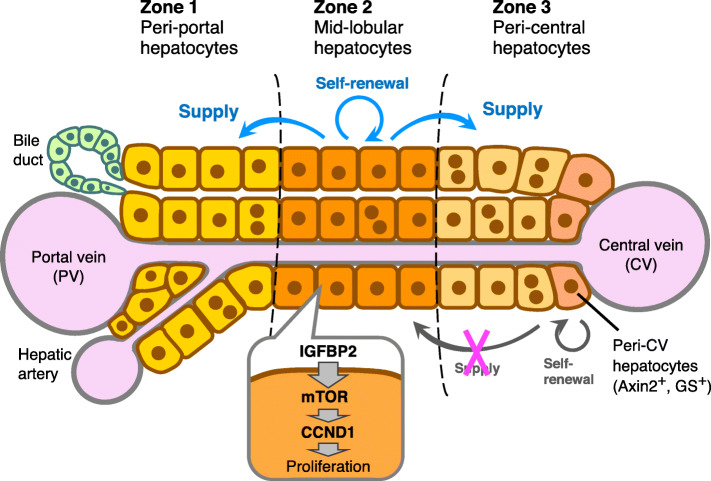
Zone 2 hepatocytes proliferate and make a predominant contribution to homeostatic maintenance of hepatocytes throughout the liver lobule. According to their division of labor, hepatocytes in the liver tissue constitute a characteristic distribution pattern known as “liver zonation”. The present studies have collectively demonstrated that zone 2 hepatocytes exhibit the highest level of proliferative activity and are the major source of hepatocyte regeneration for homeostatic tissue turnover, as well as for tissue repair in response to certain types of injury conditions, in the entire liver lobule. Proliferation of zone 2 hepatocytes is achieved in part through the IGFBP2-mTOR-CCND1 signaling axis. In zone 3, hepatocytes that locate just adjacent to the central vein (CV) are marked by the expression of Axin2 or glutamine synthetase (GS) and self-renew, but they do not contribute to hepatocyte renewal in other regions under homeostatic conditions

The liver is a versatile organ with multiple physiological functions that are essential for vital activity of the organisms, ranging from metabolism of biological macromolecules including amino acids, lipids and carbohydrates, to serum protein synthesis, detoxification of xenobiotic compounds, production and secretion of bile, and immune regulation. In order to perform these complex biological functions in parallel, the liver possesses a well-organized tissue architecture (Fig. 1). The mammalian liver lobule, which is a basic functional unit iteratively filling the organ, is typically depicted as a hexagonal or polygonal structure with the so-called central vein (CV) located in the middle and the portal triad consisting of the portal vein (PV), the hepatic artery and the bile duct at the apexes. Within the lobule, the portal blood originating from the intestine flows from the PV to the CV. Hepatocytes, the parenchymal cell-type in the liver, line up around the sinusoids connecting the portal and central veins such that they form a clear division of labor along the portal-central axis, referred to as “liver zonation”.(Ben-Moshe & Itzkovitz, 2019; Manco & Itzkovitz, 2021) There are three zones with distinct metabolic activities: hepatocytes that locate around the portal vein (zone 1) are specialized in gluconeogenesis, β-oxidation, ureageneis and cholesterol biosynthesis, while those around the central vein (zone 3) are involved in drug metabolism, glycolysis, lipogenesis, glutamine synthesis and bile acid production. Compared to these well-established functional assignments for zones 1 and 3, the physiological relevance of zone 2 that locates in between them remained largely elusive. Now, two independent studies done by Prof. Hao Zhu’s group(Wei et al., 2021) and Prof. Bin Zhou’s group,(He et al., 2021) published together in a recent issue of the journal *Science*, have elucidated an unanticipated role of zone 2 hepatocytes: they actually play a central role in the maintenance of liver mass during the homeostatic tissue turnover and in regeneration upon injury.

## Main text

While it is well recognized that many tissues such as the intestinal epithelia and the blood system rely on intrinsic tissue-specific stem cell populations for their daily renewal, the presence of such stem cells in the liver has long been enigmatic.(Itoh, 2016) Rather, a large number of studies in the last decade employing genetic lineage tracing analyses in mice based on the Cre/loxP-mediated heritable cell labeling and tracking system have convincingly demonstrated that hepatocyte renewal in mammalian livers is achieved by cell division of differentiated hepatocytes for physiological tissue turnover, and also for tissue regeneration in response to many if not all types of injury conditions.(Schaub et al., 2014; Yanger et al., 2014) Nevertheless, the nature of those hepatocytes that engage in tissue renewal still remained controversial. A study using Axin2-CreER mice that can label a subset of zone 3 hepatocytes located just around the CV has shown that those peri-CV Axin2+ hepatocytes possess a sort of “stem cell-like” feature and contribute to continuous renewal of all the hepatocytes across three zones,(Wang et al., 2015) while another study using Sox9-CreER mice have demonstrated that a specific subset of peri-portal hepatocytes marked by Sox9 expression are quiescent under physiological conditions but become activated upon liver injury to make a major contribution to parenchymal regeneration.(Font-Burgada et al., 2015) Meanwhile, other lineage tracing studies based on different Cre driver systems to label specific or randomly-selected hepatocyte subpopulations have concluded that those hepatocytes that can contribute to hepatocyte renewal are not spatially restricted but rather broadly distributed throughout the entire liver lobule.(Chen et al., 2020; Lin et al., 2018; Sun et al., 2020) A major cause of this conflicting and chaotic situation in this research field is that studies focusing on and tracing the fate of a specific subset of cells do not give information on the behavior of the other remaining cell populations, so that the presence of tissue-renewing activity in the former do not formally prove the absence of the same activity in the latter. Moreover, it should be necessary to perform side-by-side comparisons of hepatocyte subsets labeled by different Cre driver systems, as the hepatocyte behavior could be substantially influenced by experimental settings including institutional animal husbandry conditions.

To comprehensively characterize the tissue-renewing activities of hepatocytes located in each and every positions in the liver lobule, the study by Wei et al.(Wei et al., 2021) generated and employed 11 new CreER driver strains which specifically label distinct albeit sometimes overlapping subpopulations of hepatocytes along the portal-central axis. This enabled them to achieve direct comparisons of hepatocytes in different lobular locations under the same experimental conditions. Intriguingly, the authors were so attentive that they also included the Axin2-CreER and Sox9-CreER strains in their analyses to revisit the previous studies simultaneously. Through laborious lineage tracing analyses characterizing the array of differently labeled hepatocytes followed by statistical evaluations, they demonstrated that midlobular zone 2 hepatocytes are actually responsible for tissue turnover under the homeostatic conditions; the zone 2 lineage-labeled hepatocytes expanded in number and eventually repopulated zones 1 and 3, while the zone 1 and 3 hepatocytes decreased in number during homeostasis. EdU incorporation assays revealed that dividing hepatocytes were indeed enriched in midlobular zone 2 in wild-type mice, but showed aberrantly skewed distribution in zone 3 in the Axin2-CreER knock-in mice due likely to haploinsufficiency in the Axin2 gene, which nicely resolved the contradiction with the previous study employing this particular strain.(Wang et al., 2015) Lineage tracing analyses further demonstrated that zone 2 hepatocytes also contributed to regeneration in zones 1 and 3 upon chronic biliary and centrilobular injuries, respectively. Finally, the authors performed single-cell RNA sequencing analysis and CRISPR-mediated functional screening in vivo to characterize genes and pathways involved in hepatocyte proliferation, which implicated the IGFBP2-mTOR-CCND1 axis as a potentially important pathway responsible for proliferation of zone 2 hepatocytes.

Apart from taking advantage of zonally-restricted gene expression profiles, the study by He et al.(He et al., 2021) performed lineage tracing analyses with different principle of selection and labeling for hepatocytes of interest. They developed a novel and highly sophisticated system called ProTracer, where transient cell proliferation events occurring during a defined period can be cumulatively recorded in virtually any types of tissues and organs in mice. In this system, Cre/loxP-mediated labeling of proliferative cells was achieved using a variant CreER recombinase (CrexER) set under the control of a cell cycle-related gene (Ki67 or Cyclin A2) promoter. The trick to this system is that CrexER was designed such that the recombinase could revert back to a constitutively active form (i.e., the original Cre) through removal of the ER domain by way of the DreER/rox-mediated recombination. Through tamoxifen-induced transient activation of DreER, ProTracer enables continuous recording of cell proliferation after and only after the given time point when the drug was administrated. By further elaborating the system to develop hepatocyte-specific ProTracer systems, the authors characterized the proliferative status of the entire hepatocyte population in a spatially-unbiased fashion under the homeostatic and injury conditions. The results clearly demonstrated that zone 2 hepatocytes exhibited a superior proliferative capacity compared to those in zones 1 and 3, thereby making a dominant contribution to the parenchymal homeostasis under physiological conditions. With regard to tissue repair upon liver injury, zone 2 hepatocytes overall exhibited relevant contribution to parenchymal regeneration, while zone 1 and 3 hepatocytes also contributed differentially, according to the types of injury models examined.

Together, these two lineage tracing studies with orthogonal approaches for target cell selection and labeling have consistently and convincingly demonstrated the critical roles of zone 2 hepatocytes in the maintenance and regeneration of the liver parenchyma, which have been overlooked for a long time. The results and conclusions of the studies in turn raise many questions that need to be addressed for us to better understand the nature and mechanisms of tissue renewal in the liver. As the zone 2 hepatocyte descendants contribute to hepatocytes in both zone 1 and zone 3, how do cell differentiation and migration towards apparently opposite directions are controlled? The mode of action of IGFBP2, including its cognate receptor(s) expressed on zone 2 hepatocytes and how it is connected to mTOR and further to CCND1, awaits more detailed characterization. How does the IGFBP2-mTOR-CCND1 signaling axis behave upon liver injury? What are the signals alerting the presence of injury conditions in zone 1 or zone 3, and whether and how are they related to the IGFBP2-mTOR-CCND1 axis? It is known that biliary epithelial cells (BECs) also contribute to hepatocyte regeneration when the hepatocyte regenerative activity is globally compromised under extremely severe liver injury conditions.(Deng et al., 2018; Raven et al., 2017) As zone 2 hepatocytes are supposed to be least susceptible for toxic insults and may thus serve as the last bastion for hepatocyte-mediated parenchymal regeneration, it is of considerable interest to examine the possibility that inhibition of their proliferative activity could be a trigger to provoke the BEC-mediated regeneration processes.

In summary, the studies by Wei et al. and He et al. are highly appreciated in that they have succeeded in elucidating hitherto unrecognized aspects in the liver tissue organization, homeostasis and regeneration. They have made outstanding progress in our understanding of the liver biology and pathophysiology, particularly providing significant implications in the mechanistic bases for chronic liver diseases and liver tumorigenesis. Identification of the hepatocyte population with intrinsically high proliferative activity, as well as their regulatory signals, should also pave the way to accelerating the future development of hepatic organoids and artificial liver devices ex vivo for regenerative medicine and drug screening.

## Data Availability

Not applicable.
